# Taxonomy of *Fissocantharis* Pic (Coleoptera, Cantharidae) from Guangxi, China, with descriptions of six new species

**DOI:** 10.3897/zookeys.489.9153

**Published:** 2015-03-23

**Authors:** Yuxia Yang, Limei Li, Kaile Guan, Xingke Yang

**Affiliations:** 1 College of Life Sciences, Hebei University, Baoding 071002, Hebei Province, China; 2 Key Laboratory of Zoological Systematics and Evolution, Institute of Zoology, Chinese Academy of Sciences, Beijing 100101, China

**Keywords:** Taxonomy, Cantharidae, *Fissocantharis*, new species, synonym, Guangxi, China

## Abstract

A total of 17 species of *Fissocantharis* Pic is recorded from Guangxi, China. Six species are described new to science, *Fissocantharis sinensomima* **sp. n.**, *Fissocantharis sexcostata* **sp. n.**, *Fissocantharis basilaris* **sp. n.**, *Fissocantharis eschara* **sp. n.**, *Fissocantharis latipalpa* **sp. n.** and *Fissocantharis biprojicientis* **sp. n.**, and two previously known species are redescribed, *Fissocantharis gracilipes* (Pic, 1927) and *Fissocantharis sinensis* (Wittmer, 1988). These species are presented with habitus of males, abdominal sternites VIII of females and genitalia of both sexes. *Fissocantharis flavofacialis* (Pic, 1926) is synonymized with *Fissocantharis angusta* (Fairmaire, 1900); both were originally described in the genus *Podabrus* Westwood. Additionally, a key and a checklist of all the species of *Fissocantharis* from Guangxi are provided.

## Introduction

The species of *Fissocantharis* Pic, 1921 (redefined by Yang et al. 2009) are widely distributed in the Oriental and Palaearctic Regions. In China, about 90 species of this genus have been known until now, and their descriptions or revisions were mostly contributed by Wittmer (1951, 1972, 1979, 1982, 1983, 1988, 1989, 1993, 1995, 1997). During our study, 6 new species from Guangxi Zhuang Autonomous Region are recently discovered, and they are described here under the names of *Fissocantharis sinensomima* sp. n., *Fissocantharis sexcostata* sp. n., *Fissocantharis basilaris* sp. n., *Fissocantharis eschara* sp. n., *Fissocantharis latipalpa* sp. n. and *Fissocantharis biprojicientis* sp. n. For some comparisons with the new species, *Fissocantharis gracilipes* (Pic, 1927) and *Fissocantharis sinensis* (Wittmer, 1988) are redescribed and provided with some supplementary characters.

*Fissocantharis flavofacialis* (Pic, 1926) is considered to be a junior synonym of *Fissocantharis angusta* (Fairmaire, 1900), which were both originally described in *Podabrus* Westwood, 1838 from Fujian, China, since no differences are found between them. A key and a checklist of all species from Guangxi are presented, as well as some additional distributional data are provided for some previously known species.

## Material and methods

The material is preserved in the following collections. Primary types are returned to the collections from which they are borrowed or are otherwise deposited in public museums.

IZAS Institute of Zoology, Chinese Academy of Sciences, Beijing, China;

MHBU Museum of Hebei University, Baoding, China;

MNHN Muséum national d’Histoire naturelle, Paris, France;

NHMB Naturhistorisches Museum Basel, Switzerland;

ZFMK Zoologische Forschungsinstitut und Museum “Alexander Koenig”, Bonn, Germany.

The genitalia of both sexes and abdominal sternites VIII of females are dissected and cleared in 10% KOH solution, and the female genitalia is dyed with hematoxylin. Habitus photos are taken by a Leica M205 A microscope, multiple layers are stacked using Combine ZM (Helicon Focus 5.3). Line drawings are made with the aid of camera lucida attached to a Leica MZ12.5 stereomicroscope, then edited in CorelDRAW 12 and Adobe Photoshop 8.0.1.

Complete label data are cited for type specimens, quotation marks are used to separate data from different labels and a backslash “\” to separate data from different lines of the same label.

Body length is measured from the anterior margin of the clypeus to the elytral apex and body width across the humeral part of elytra. Morphological terminology of female genitalia follows that of Brancucci (1980). The abbreviations in the figures are as follows, ag: accessory gland; di: diverticulum; sd: spermathecal duct; sp: spermatheca; ov: median oviduct; va: vagina.

## Taxonomy

### Key to the species of *Fissocantharis* Pic in male from Guangxi, China

**Table:** 

1	Middle antennomeres strongly deformed	**2**
–	Antennae filiform or middle antennomeres slightly flattened or thickened	**10**
2	Antennomeres III‒IV or V deformed, others normal	**3**
–	Antennomeres III‒XI deformed	**5**
3	Head mostly black; antennomeres III‒V deformed and maxillary palpomeres II‒III normal	***Fissocantharis tridifformis* (Wittmer, 1988)**
–	Head uniformly orange; antennomeres III‒IV deformed, V normal and maxillary palpomeres II‒III deformed	**4**
4	Antennomeres IV with two projections at basal part; maxillary palpomeres II‒III excavated wholly on dorsal sides	***Fissocantharis biprojicientis* sp. n.**
–	Antennomeres IV unlike above, without projections; maxillary palpomeres II‒III each with a deep round pit on dorsal side	***Fissocantharis bidifformis* (Wittmer, 1988)**
5	Antennomeres III‒VIII each emarginated at apical part of outer margin	***Fissocantharis multiexcavata* (Wittmer, 1988)**
–	Antennomeres III‒VIII unlike above	**6**
6	Antennomeres thickened, nearly parallel-sided	**7**
–	Antennomeres flattened and widened apically	**8**
7	Antennomeres VIII with outer apical angles strongly projecting laterad, III‒VIII minutely serrated along outer margins	***Fissocantharis flavicornis* (Gorham, 1889)**
–	Antennomeres VII‒VIII with outer apical angles moderately projecting laterad, III‒VIII not serrated	***Fissocantharis cicatricosa* (Wittmer, 1988)**
8	Antennomeres X shortened, XI widened near base, knife-like	**9**
–	Antennomeres X and XI normal, parallel-sided	***Fissocantharis liuchowensis* (Wittmer, 1989)**
9	Antennomeres XI about one-third longer than X	***Fissocantharis angusta* (Fairmaire, 1900)**
–	Antennomeres XI about as twice long as X	***Fissocantharis tachulanensis* (Wittmer, 1988)**
10	Middle antennomeres with longitudinal ridges along outer margins	**11**
–	Middle antennomeres unlike above	**12**
11	Antennae slightly thickened, antennomeres III‒IX with longitudinal ridges along outer margins; aedeagus: conjoint dorsal plate of parameres well-developed, distinctly longer than ventral processes	***Fissocantharis buonloiensis* Wittmer, 1993**
–	Antennae slightly flattened, antennomeres III‒VIII with longitudinal ridges along outer margins; aedeagus: conjoint dorsal plate of parameres moderately reduced, distinctly shorter than ventral processes	***Fissocantharis sexcostata* sp. n.**
12	Maxillary palpomeres II‒IV flattened and widened, II convex at basal part of dorsal side; pronotum uniformly black	***Fissocantharis latipalpa* sp. n.**
–	Maxillary palpi normal; pronotum uniformly orange or mixed with black marking	**13**
13	Antennomeres IV‒XI each with an oblong smooth scar-like bulge on outer margin	**14**
–	Antennomeres IV‒XI unlike above	**15**
14	Body larger, more than 9.0 mm in length; aedeagus: conjoint dorsal plate of parameres greatly reduced, slightly roundly protuberant in middle of apical margin, ventral process of each paramere abruptly narrowed apically, slightly hooked at apex	***Fissocantharis gracilipes* (Pic, 1927)**
–	Body smaller, less than 9.0 mm in length; aedeagus: conjoint dorsal plate of parameres moderately reduced, tapered at apical margin, ventral process of each paramere evenly narrowed apically, moderately hooked at apex	***Fissocantharis eschara* sp. n.**
15	Antennomeres III‒X parallel-sided, IV‒XI each with a narrow smooth longitudinal impression at basal part of outer margin	***Fissocantharis sinensomima* sp. n.**
–	Antennomeres III‒X slightly flattened and obliquely widened apically, IV‒XI unlike above	**16**
16	Antennomeres V‒VIII each with a longitudinal smooth impression at apical part of outer margin	***Fissocantharis sinensis* (Wittmer, 1988)**
–	Antennomeres IV‒XI each with a round smooth impression at base of outer margin	***Fissocantharis basilaris* sp. n.**

### Description of the species

#### Fissocantharis sinensis

Taxon classificationAnimaliaColeopteraCantharidae

(Wittmer, 1988)

**Figure 1.**
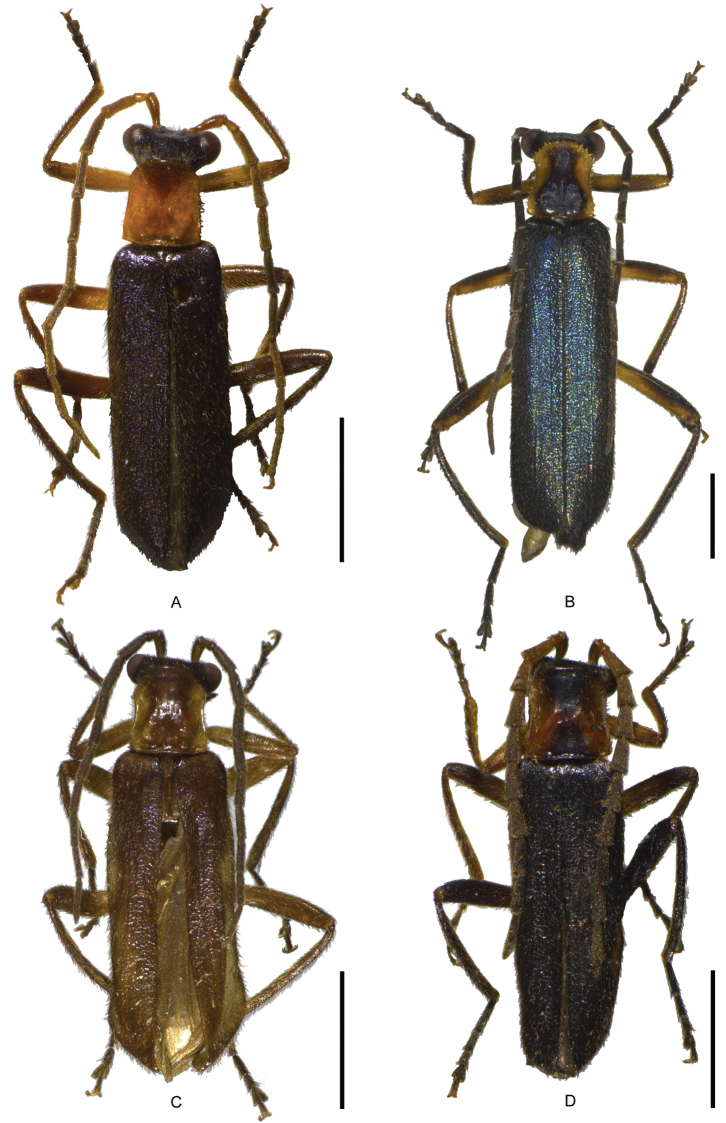
Male habitus, dorsal view: **A** *Fissocantharis sinensis* (Wittmer, 1988) **B** *Fissocantharis gracilipes* (Pic, 1927) **C** *Fissocantharis sinensomima* sp. n. **D** *Fissocantharis sexcostata* sp. n. Scale bars: 2.0 mm.

Figs 1A , 3A‒C , 8A , 9A

Micropodarus sinensis Wittmer, 1988: 353, figs 8, 28.
Fissocantharis sinensis : Yang et al. 2009: 49.

##### Type material examined.

Holotype: 1♂ (IZAS): “阳朔26.IV938” [Guangxi: Yangshuo], “Micropodabrus \ sinensis \ Wittm. \ det. W. Wittmer”, “HOLOTYPUS”.

##### Additional material examined.

CHINA: Guangxi: 4♂♂, 1♀ (IZAS): Lingchuan, 6.‒7.VI.1984, collector unknown; 1♂ (IZAS): Xing’an, 210m, 1.VI.1984, collector unknown; 1♀ (IZAS): Yangshuo, 29.IV.1938, collector unknown; 1♂ (IZAS): Beiquan, 29.V.1939, collector unknown.

##### Redescription.

Male (Fig. 1A). Head black, mouthparts blackish brown, light brown at bases of mandibles and labium, antennae black, yellow at ventral sides of antennomeres I‒II, prothorax orange, pronotum sometimes with a large black marking in middle of disc, which extending from anterior to posterior margin, scultellum black, elytra dark purple, with weak metallic shine, legs black, yellow at pro-coxae, trochanters and basal parts of femora, meso- and metasterna and abdomen black. Body densely covered with short decumbent light brown pubescence, also mixed with slightly long semierect pubescence along anterior margin of labrum and on disc of elytra.

Head subquadrate, temples evenly narrowed posteriad, surface semilustrous, finely and densely punctate; eyes strongly protruding, head breadth across eyes distinctly wider than anterior margin of pronotum; maxillary palpomeres IV longer than wide, widest at apical one-third, arcuate and sharp at apical parts of inner margins; antennae almost extending to apical one-third length of elytra, antennomeres II slightly longer than wide at apices, III‒X slightly flattened and obliquely widened apically, III about twice as long as II, IV slightly longer than VIII, V‒VIII each with a longitudinal smooth impression at apical part of outer margin, XI parallel-sided, slightly longer than X and pointed at apices.

Pronotum about 1.10 times longer than wide, widest near base, anterior margin rounded, anterior angle rounded, lateral margins sinuate, slightly diverging posteriad, posterior angle nearly rectangular, posterior margin arcuate and slightly bordered, disc distinctly convex on postero-lateral parts, surface semilustrous, punctate like that on head.

Elytra about 3.7 times longer than pronotum, 2.8 times longer than humeral width, lateral margins nearly parallel, disc surface semilustrous, rugulose-lacunose and finely punctate.

All tarsal claws bifid, upper claws nearly as long as lower claws.

Aedeagus (Fig. 3A‒C): conjoint dorsal plate of parameres well-developed, about half length of ventral processes, nearly parallel-sided, with apical margin tapered apically; ventral process of each paramere evenly narrowed apically, largely hooked at apex.

Female. Similar to male, but eyes not so protruding; antennae shorter, extending to elytral mid-length, antennomeres III‒X nearly parallel-sided, V‒VIII without impressions; pronotum slightly wider, about 1.05 times longer than wide, moderately convex at posterolateral parts of disc; legs black at profemora. Abdominal sternite VIII (Fig. 8A) slightly emarginated on both sides of posterior margin, middle part between lateral emarginations slightly acute at apex, latero-apical angles widely rounded. Internal organ of reproductive system (Fig. 9A): vagina stout and abruptly narrowed and extended into a long duct above median oviduct; diverticulum and spermathecal duct arising from the end of the long duct of vagina; diverticulum moderately long, thin and spiral; spermathecal duct distinctly thicker and shorter than diverticulum; spermatheca composed of a spiral tube which is distinctly longer than diverticulum, provided with a very long and thin accessory gland which is much longer than the spiral tube of spermatheca; median oviduct situated in middle of vagina.

Body length: 6.5‒8.0 mm; width: 1.2‒1.7 mm.

##### Distribution.

China (Guangxi, Sichuan).

##### Remarks.

In the original publication (Wittmer 1988), some characteristics of antennae for the male is not indicated, which however is important for diagnosis of *Fissocantharis* species. Herein it is redescribed and also provided with some supplementary characters for abdominal sternite VIII and genitalia of the female.

#### Fissocantharis gracilipes

Taxon classificationAnimaliaColeopteraCantharidae

(Pic, 1927)

Figs 1B , 3D‒F , 8B , 9B

Fissopodabrus gracilipes Pic, 1927: 2.
Micropodabrus gracilipes : Wittmer 1982: 127; 1988: 351, figs 5, 24, 25.
Fissocantharis gracilipes : Yang et al. 2009: 49.

##### Type material examined.

Holotype: 1♂ (MNHN): [p]“Tonkin \ Chapa\ 3.V.1918 \ Jeanvoine”, [h]“Fissopodabrus \ gracilipes n. sp.”, [h]“Micropodabrus \ gracilipes \ (Pic) \ det. W. Wittmer”, [p]“TYPE”.

##### Additional material examined.

1♂, 1♀ (MHBU): CHINA: Guangxi, Wuming, Damingshan, 600‒900m, 25.V.2011, leg. H.Y. Liu; 2♂♂ (MHBU): same locality and collector, 27.V.2011, 1100m; 2♀♀ (MHBU): same locality and collector, 20.V.2011, 1230‒1423m.

##### Redescription.

Male (Fig. 1B). Head black, mouthparts blackish brown, light brown at bases of mandibles and labium, antennae black, prothorax yellow, pronotum with a large blackish brown marking in middle of disc, which extending nearly from anterior to posterior margin, scultellum black, elytra blue, with strong metallic shine, legs black, yellow at coxae, trochanters and ventral sides of femora and tibiae, meso- and metasterna and abdomen black. Body densely covered with short decumbent dark brown pubescence, also mixed with slightly long semierect pubescence along anterior margin of labrum and on disc of elytra.

Head subquadrate, temples evenly narrowed posteriad, surface semilustrous, finely and densely punctate; eyes strongly protruding, head breadth across eyes distinctly wider than anterior margin of pronotum; maxillary palpomeres IV longer than wide, widest at apical one-third, arcuate and sharp at apical parts of inner margins; antennae filiform, almost extending to apical one-fourth length of elytra, antennomeres II slightly longer than wide at apices, III about twice as long as II, IV slightly longer than III, IV‒XI each with an oblong smooth scar-like bulge at basal part of outer margin, XI slightly longer than X and pointed at apices.

Pronotum about 1.17 times longer than wide, widest near base, anterior margin rounded, anterior angle rounded, lateral margins sinuate, moderately diverging posteriorly, posterior angle nearly rectangular, posterior margin arcuate and slightly bordered, disc distinctly convex on posterolateral parts, surface semilustrous, sparsely and finely punctate.

Elytra about 4.0 times longer than pronotum, 3.3 times longer than humeral width, lateral margins nearly parallel, disc surface semilustrous, rugulose-lacunose and finely punctate.

All tarsal claws bifid, upper claws nearly as long as lower claws.

Aedeagus (Fig. 3D‒F): conjoint dorsal plate of parameres greatly reduced, slightly roundly protuberant in middle of apical margin; ventral process of each paramere abruptly narrowed apically, slightly hooked at apex.

Female. Similar to male, but eyes not so protruding; antennae shorter, extending to elytral mid-length, antennomeres IV‒XI without bulges; pronotum slightly wider, about 1.10 times longer than wide, moderately convex at posterolateral parts of disc. Abdominal sternite VIII (Fig. 8B) slightly emarginated on both sides of posterior margin, middle part between lateral emarginations arcuate, latero-apical angles widely rounded. Internal organ of reproductive system (Fig. 9B): vagina stout and abruptly narrowed and extended into a long duct above median oviduct; diverticulum and spermathecal duct arising from the end of the long duct of vagina; diverticulum slightly long, thin and spiral; spermathecal duct distinctly thicker and nearly as long as diverticulum; spermatheca composed of a spiral tube which is distinctly longer than diverticulum, provided with a very long and thin accessory gland which is much longer than the spiral tube of spermatheca; median oviduct situated in middle of vagina.

Body length: 9.0‒12.0 mm; width: 1.5‒2.5 mm.

##### Distribution.

China (new country record: Guangxi); Vietnam.

##### Remarks.

The elytra of the holotype are purple, but the coloration could be variable in cantharid species bearing a metallic shine, not only in *Fissocantharis*, but also in *Themus* Motschulsky. By contrast, the characteristics of the aedeagus and antennae of the male are much more stable and reliable, which are the basis of our determination of the additional specimens as this species.

#### Fissocantharis sinensomima

Taxon classificationAnimaliaColeopteraCantharidae

Y. Yang & X. Yang sp. n.

http://zoobank.org/7C83317C-5AEB-4152-BDDA-3CD1CC466459

Figs 1C , 4A‒C

##### Type material.

Holotype ♂ (IZAS): CHINA: Guangxi, Napo, Nonghua, 1000m, 14.IV.1998, leg. C.S. Wu.

##### Description.

Male (Fig. 1C). Head black, mouthparts blackish brown, light brown at bases of mandibles and labium, antennae black, yellow at ventral sides of antennomeres I‒II, prothorax yellow, pronotum with a large black marking in middle of disc, which extending from anterior to posterior margin, scultellum black, elytra dark purple, with weak metallic shine, legs black, yellow at coxae, trochanters and basal parts of femora, meso- and metasterna and abdomen black. Body densely covered with short decumbent light brown pubescence, also mixed with slightly long semierect pubescence along anterior margin of labrum and on disc of elytra.

Head subquadrate, temples evenly narrowed posteriorly, surface semilustrous, finely and densely punctate; eyes strongly protruding, head breadth across eyes distinctly wider than anterior margin of pronotum; maxillary palpomeres IV longer than wide, widest at apical one-third, arcuate and sharp at apical parts of inner margins; antennae filiform, almost extending to apical one-third length of elytra, antennomeres II slightly longer than wide at apices, III‒XI parallel-sided, III about twice as long as II, IV‒XI each with a narrow longitudinal smooth impression at basal part of outer margin, IV about one-third longer than III, XI slightly longer than X and pointed at apices.

Pronotum about 1.10 times longer than wide, widest near base, anterior margin rounded, anterior angle rounded, lateral margins sinuate, slightly diverging posteriad, posterior angle nearly rectangular, posterior margin arcuate and slightly bordered, disc distinctly convex on posterolateral parts, surface semilustrous, punctate like that on head.

Elytra about 3.7 times longer than pronotum, 3.0 times longer than humeral width, lateral margins nearly parallel, disc surface semilustrous, rugulose-lacunose and finely punctate.

All tarsal claws bifid, upper claws nearly as long as lower claws.

Aedeagus (Figs 4A‒C): conjoint dorsal plate of parameres greatly reduced, slightly emarginated in middle of apical margin; ventral process of each paramere evenly narrowed apically, largely hooked at apex.

Female. Unknown.

Body length: 6.0 mm; width: 1.5 mm.

##### Diagnosis.

This species is similar to *Fissocantharis sinensis*, but can be distinguished by the antennomeres IV‒XI each with a narrow longitudinal smooth impression along basal part of outer margin in male; aedeagus: conjoint dorsal plate of parameres greatly reduced, slightly emarginated in middle of apical margin.

##### Distribution.

China (Guangxi).

##### Etymology.

The specific name is derived from Latin *mimus* (similar, imitating something), referring to its similarity to *Fissocantharis sinensis* (Wittmer, 1988).

#### Fissocantharis sexcostata

Taxon classificationAnimaliaColeopteraCantharidae

Y. Yang & X. Yang sp. n.

http://zoobank.org/CF675FDA-2F8C-44E8-960B-DB392B95F153

Figs 1D , 4D‒F , 8C , 9C

##### Type material.

Holotype ♂ (IZAS): CHINA: Guangxi, Jinxiu, Huawangshanzhuang, 600m, 20.V.1999, leg. M.Y. Gao. Paratypes: 1♂, 2♀♀ (IZAS): same data as the holotype; 2♂♂ (IZAS): same locality and date, leg. Y.Z. Zhang; 1♂ (IZAS): same locality and date, leg. H. Xiao; 1♀ (IZAS): same locality and date, leg. W. Z. Li; 1♂ (IZAS): same locality and date, leg. H.X. Han; 1♀ (IZAS): same locality and date, leg. X.K. Li; 1♀ (IZAS): same locality and date, leg. D.C. Yuan.

##### Description.

Male (Fig. 1D). Head black, mouthparts blackish brown, light brown at bases of mandibles and labium, antennae black, orange at antennomeres I‒II and ventral sides of III, prothorax orange, pronotum with a large inverse-triangular and a slightly small triangular black markings in middle of anterior and posterior parts of disc respectively, two markings almost conjoint, scultellum black, elytra dark purple, with weak metallic shine, legs black, yellow at pro-coxae, trochanters and femora and meso-trochanters and bases of femora, meso- and metasterna and abdomen black. Body densely covered with short decumbent light brown pubescence, also mixed with slightly long semierect pubescence along anterior margin of labrum and on disc of elytra.

Head subquadrate, temples evenly narrowed posteriorly, surface semilustrous, finely and densely punctate; eyes moderately protruding, head breadth across eyes distinctly wider than anterior margin of pronotum; maxillary palpomeres IV longer than wide, widest at apical one-third, arcuate and sharp at apical parts of inner margins; antennae almost extending to apical one-third length of elytra, antennomeres II nearly as long as wide at apices, III‒X slightly widened apically, nearly long-triangular, the whole length of III‒VII and basal two-thirds length of VIII each with a longitudinal ridge along outer margin, IV slightly longer than III, XI parallel-sided, slightly longer than X and pointed at apices.

Pronotum about 1.10 times longer than wide, widest near base, anterior margin rounded, anterior angle rounded, lateral margins slightly sinuate and diverging posteriad, posterior angle nearly rectangular, posterior margin arcuate and slightly bordered, disc distinctly convex on posterolateral parts, surface semilustrous, punctate like that on head.

Elytra about 3.4 times longer than pronotum, 3.0 times longer than humeral width, lateral margins nearly parallel, disc surface semilustrous, rugulose-lacunose and finely punctate.

All tarsal claws bifid, upper claws nearly as long as lower claws.

Aedeagus (Fig. 4D‒F): conjoint dorsal plate of parameres moderately reduced, distinctly shorter than ventral process, with apical margin tapered apically; ventral process of each paramere evenly narrowed apically, largely hooked at apex.

Female. Similar to male, but eyes not so protruding; antennae uniformly black, antennomeres III‒X nearly parallel-sided, III‒VIII without ridges; pronotum slightly wider, about 1.12 times longer than wide, lateral margins sinuate, moderately diverging posteriad, moderately convex at posterolateral parts of disc, legs orange at pro-coxae and trochanters. Abdominal sternite VIII (Fig. 8C) slightly emarginated on both sides of posterior margin, middle part between lateral emarginations slightly arcuate, latero-apical angles narrowly rounded. Internal organ of reproductive system (Fig. 9C): vagina stout and abruptly narrowed and extended into a long duct above median oviduct; diverticulum and spermathecal duct arising from the end of the long duct of vagina; diverticulum slightly long, thin and spiral; spermathecal duct distinctly thicker and shorter than diverticulum; spermatheca composed of a spiral tube which is distinctly longer than diverticulum, provided with a long and thin accessory gland which is slightly longer than the spiral tube of spermatheca; median oviduct situated in middle of vagina.

Body length: 6.0‒10.0 mm; width: 1.2‒2.0 mm.

##### Diagnosis.

This species is similar to *Fissocantharis sinensis*, but can be easily differentiated by the antennomeres III‒VIII with longitudinal ridges along outer margins in male; aedeagus: conjoint dorsal plate of parameres moderately reduced.

##### Distribution.

China (Guangxi).

##### Etymology.

The specific name is derived from Latin *sex*- (six) and *costatus* (ridged), referring to its antnnomeres III‒VIII with longitudinal ridges (six ridges in total) along outer margins in male.

##### Remarks.

Sometimes the pronotum is uniformly orange, without any black markings, and this variation always occurs on the females.

#### Fissocantharis basilaris

Taxon classificationAnimaliaColeopteraCantharidae

Y. Yang & X. Yang sp. n.

http://zoobank.org/397D3015-0CA8-4805-B017-DEBC7BBCC54F

**Figure 2.**
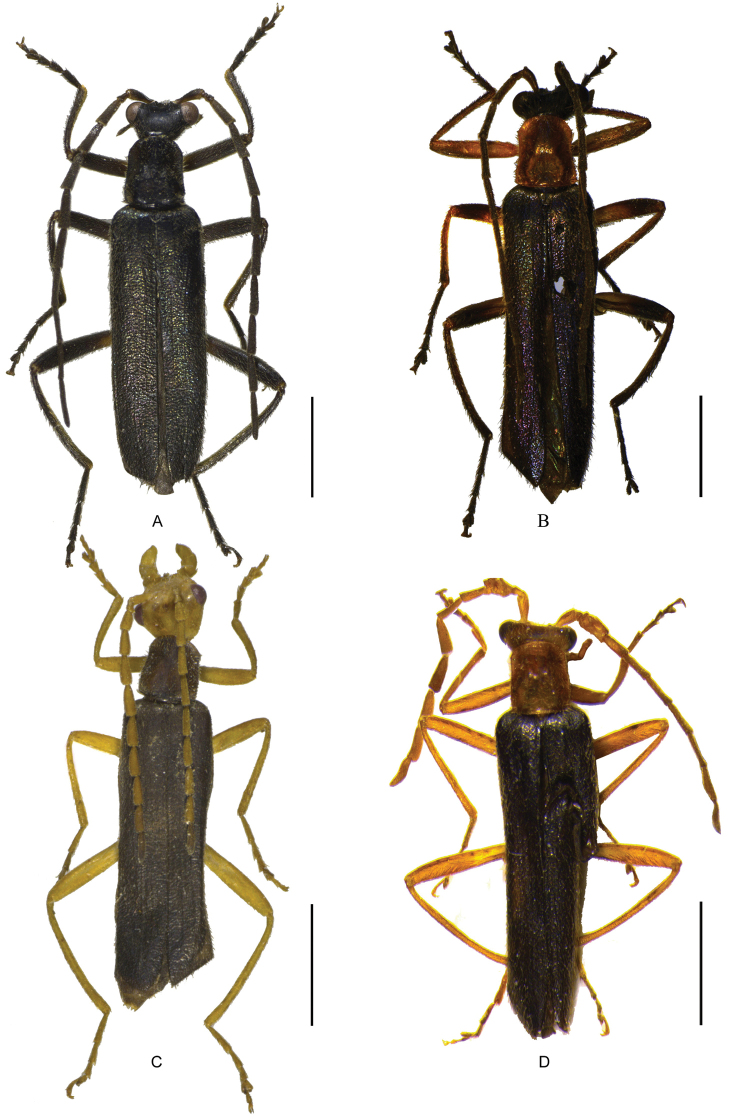
Male habitus, dorsal view: **A** *Fissocantharis basilaris* sp. n. **B** *Fissocantharis eschara* sp. n. **C** *Fissocantharis latipalpa* sp. n. **D** *Fissocantharis biprojicientis* sp. n. Scale bars: 2.0 mm.

Figs 2A , 5A‒C , 8D , 10A

Fissocantharis langaniformis (Wittmer, 1989): Yang et al. 2014: 14 [misidentification].

##### Type material.

Holotype ♂ (MHBU): CHINA: Guangxi: Wuming, Damingshan, 1100m, 27.V.2011, leg. H.Y. Liu. Paratypes: CHINA: Guangxi: 26♂♂, 17♀♀ (MHBU): same data to the holotype; 20♂♂, 13♀♀ (MHBU): same locality and collector, 1230‒1423m, 20.V.2011; 4♂♂, 4♀♀ (MHBU): same locality and collector, 600–900m, 25.V.2011; 1♀ (MHBU): same locality, 23.V 2011, leg. Li-Ying Guo.

##### Description.

Male (Fig. 2A). Head black, mouthparts blackish brown, light brown at bases of mandibles and labium, antennae black, yellow at ventral sides of antennomeres I‒III, pronotum black, scultellum black, elytra blue, with strong metallic shine, legs black, yellow at apical parts of coxae, trochanters and basal parts of femora, presternum dark brown, meso- and metasterna and abdomen black. Body densely covered with short decumbent dark brown pubescence, also mixed with slightly long semierect pubescence along anterior margin of labrum and on disc of elytra.

**Figures 3.**
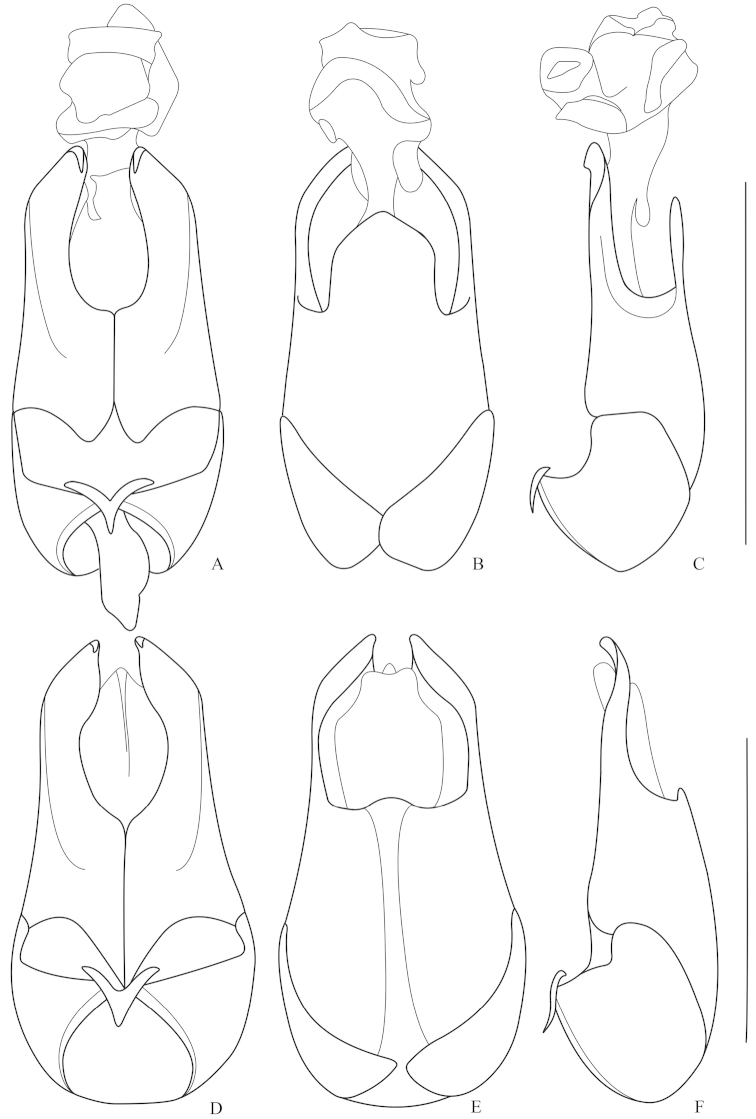
Aedeagus (**A, D** ventral view **B, E** dorsal view **C, F** lateral view): **A–C** *Fissocantharis sinensis* (Wittmer, 1988) **D–F** *Fissocantharis gracilipes* (Pic, 1927). Scale bars: 1.0 mm.

**Figures 4.**
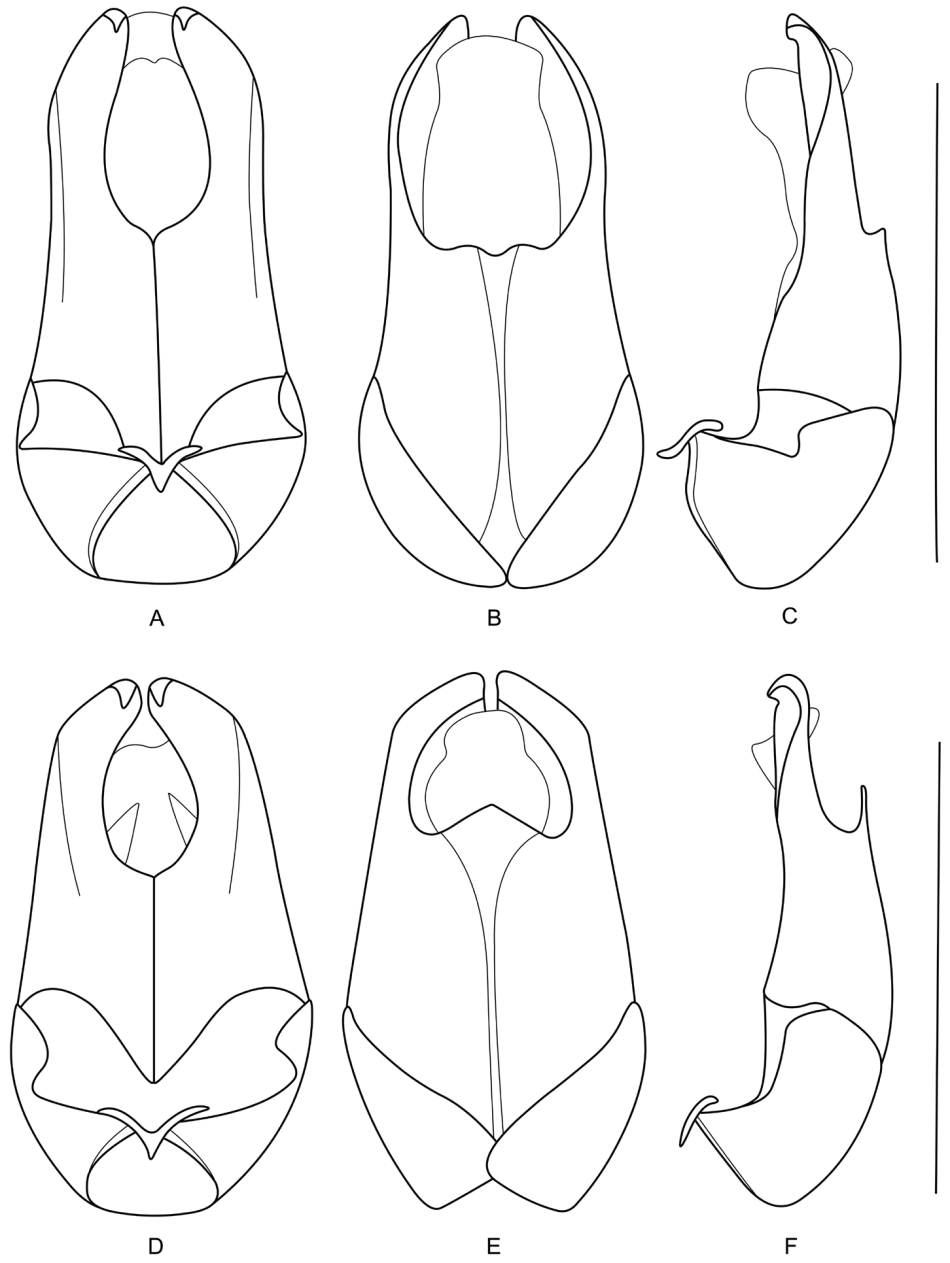
Aedeagus (**A, D** ventral view **B, E** dorsal view **C, F** lateral view): **A–C** *Fissocantharis sinensomima* sp. n. **D–F** *Fissocantharis sexcostata* sp. n. Scale bars: 1.0 mm.

Head subquadrate, temples evenly narrowed posteriad, surface semilustrous, finely and densely punctate; eyes strongly protruding, head breadth across eyes distinctly wider than anterior margin of pronotum; maxillary palpomeres IV longer than wide, widest at apical one-third, arcuate and sharp at apical parts of inner margins; antennae almost extending to apical one-fourth length of elytra, antennomeres II slightly longer than wide at apices, III‒X slightly flattened and widened apically, III about twice as long as II, IV‒XI each with a small rounded smooth impression at base of outer margin, IV about one-third longer than III, XI nearly parallel-sided, slightly longer than X and pointed at apices.

Pronotum about 1.26 times longer than wide, widest near base, anterior margin rounded, anterior angle rounded, lateral margins sinuate, moderately diverging posteriorly, posterior angle nearly rectangular, posterior margin arcuate and slightly bordered, disc distinctly convex on posterolateral parts, surface semilustrous, punctate like that on head.

Elytra about 4.0 times longer than pronotum, 3.0 times longer than humeral width, lateral margins nearly parallel, disc surface semilustrous, rugulose-lacunose and finely punctate.

All tarsal claws bifid, upper claws nearly as long as lower claws.

**Figures 5.**
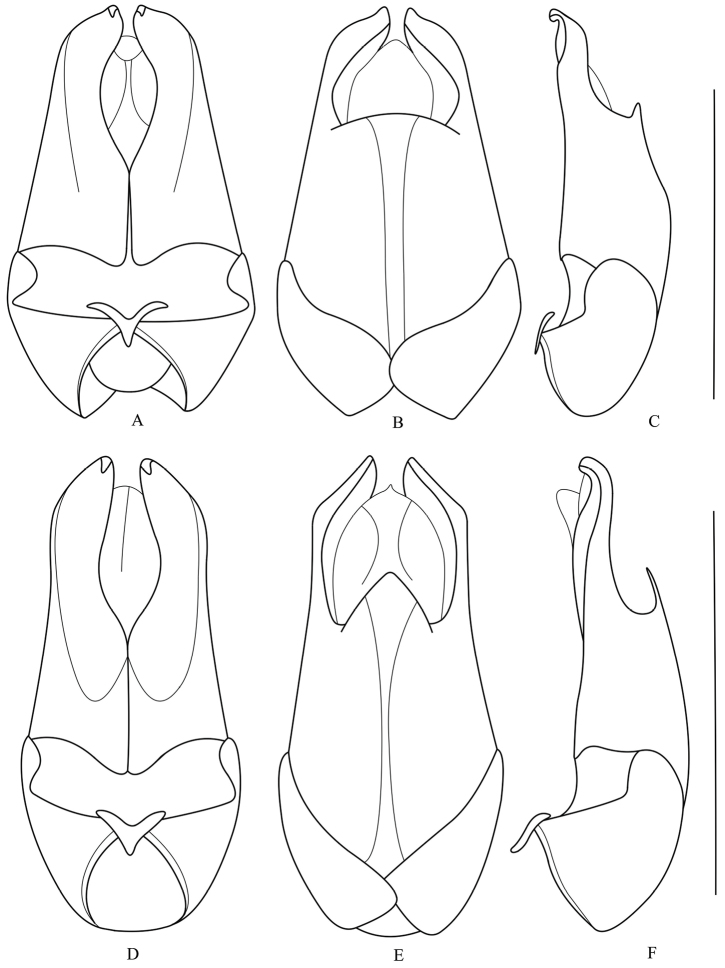
Aedeagus (**A, D** ventral view **B, E** dorsal view **C, F** lateral view): **A–C** *Fissocantharis basilaris* sp. n. **D–F** *Fissocantharis eschara* sp. n. Scale bars: 1.0 mm.

Aedeagus (Fig. 5A‒C): conjoint dorsal plate of parameres greatly reduced, rounded at apical margin; ventral process of each paramere evenly narrowed apically, slightly hooked at apex.

Female. Similar to male, but eyes not so protruding; antennae shorter, extending to elytral mid-length, antennomeres III‒X nearly parallel-sided, IV‒XI without impressions; pronotum slightly wider, about 1.13 times longer than wide, moderately convex at postero-lateral parts of disc. Abdominal sternite VIII (Fig. 8D) roundly protuberant in middle of posterior margin, latero-apical angels subrounded. Internal organ of reproductive system (Fig. 10A): vagina stout and abruptly narrowed and extended into a long duct above median oviduct; diverticulum and spermathecal duct arising from the end of the long duct of vagina; diverticulum slightly long, thin and spiral; spermathecal duct distinctly thicker and slightly shorter than diverticulum; spermatheca composed of a spiral tube which is distinctly longer than diverticulum, provided with a very long and thin accessory gland, which is much longer than the spiral tube of spermatheca; median oviduct situated in middle of vagina.

Body length: 7.0‒11.0 mm; width: 1.2‒2.0 mm.

##### Diagnosis.

This species is similar to *Fissocantharis langaniformis* (Wittmer, 1989), but can be distinguished from the latter by the antennomeres IV‒XI each with a rounded smooth impression at base of outer margin in male; aedeagus: conjoint dorsal plate of parameres rounded at apical margin.

##### Distribution.

China (Guangxi).

##### Etymology.

The specific name is derived from Latin *basilaris* (basal), referring to its antennomeres IV‒XI each with a rounded impression at base of outer margin in male.

#### Fissocantharis eschara

Taxon classificationAnimaliaColeopteraCantharidae

Y. Yang & X. Yang sp. n.

http://zoobank.org/741C5C3E-BE67-4F1B-8459-AF80116E3C7A

Figs 2B , 5D‒F , 8E , 10B

##### Type material.

Holotype ♂ (IZAS): CHINA: Guangxi: Jinxiu, Rd. Jinzhong, 1100m, 11.V.1999, leg. D.C. Yuan. Paratypes: CHINA: Guangxi: 1♂ (IZAS): same locality as the holotype, 12.V.1999, leg. W.Z. Li; 1♀ (IZAS): same locality, 12.V.1999, leg. X.K. Yang; 1♀ (IZAS): same locality, 10.V.1999, leg. X.K. Yang; 1♀ (IZAS): same locality, 1000m, 10.V.1999, leg. F.S. Huang; 1♂ (IZAS): same locality, 1000m, 12.V.1999, leg. X.Z. Zhang; 2♀♀ (IZAS): same locality, 1000m, 12.V.1999, leg. M.Y. Gao; 1♂, 1♀ (IZAS): Jinxiu, Fenzhan, 13.V.1999, leg. H. Xiao; 1♂ (IZAS): Jinxiu, Luoxiang, 400m, 15.V.1999, leg. D.C. Yuan; 1♀ (IZAS): same data, leg. D.J. Liu; 1♀ (IZAS): same locality and date, 200m, leg. X.Z. Zhang.

##### Description.

Male (Fig. 2B). Head black, mouthparts blackish brown, light brown at bases of mandibles and labium, antennae black, yellow at ventral sides of antennomeres I‒II, prothorax orange, scultellum black, elytra dark purple, with weak metallic shine, legs black, yellow at coxae, trochanters and basal parts of femora, meso- and metasterna and abdomen black. Body densely covered with short decumbent light brown pubescence, also mixed with slightly long semierect pubescence along anterior margin of labrum and on disc of elytra.

Head subquadrate, temples evenly narrowed posteriorly, surface semilustrous, finely and densely punctate; eyes strongly protruding, head breadth across eyes distinctly wider than anterior margin of pronotum; maxillary palpomeres IV longer than wide, widest at apical one-third, arcuate and sharp at apical parts of inner margins; antennae filiform, nearly extending to elytral apices, antennomeres II slightly longer than wide at apices, III about twice as long as II, IV‒XI each with an oblong smooth scar-like bulge at basal part of outer margin, IV slightly longer than III, XI slightly shorter than X and pointed at apices.

Pronotum about 1.29 times longer than wide, widest near base, anterior margin rounded, anterior angle rounded, lateral margins slightly sinuate and diverging posteriad, posterior angle nearly rectangular, posterior margin arcuate and slightly bordered, disc distinctly convex on posterolateral parts, surface semilustrous, punctate like that on head.

Elytra about 4.0 times longer than pronotum, 3.0 times longer than humeral width, lateral margins nearly parallel, disc surface semilustrous, rugulose-lacunose and finely punctate.

All tarsal claws bifid, upper claws nearly as long as lower claws.

Aedeagus (Fig. 5D‒F): conjoint dorsal plate of parameres moderately reduced, distinctly shorter than ventral processes, with apical margin tapered apically; ventral process of each paramere evenly narrowed apically, moderately hooked at apex.

Female. Similar to male, but eyes not so protruding; antennae uniformly black, antennomeres IV‒XI without scar-like bulges; pronotum slightly wider, about 1.13 times longer than wide, lateral margins sinuate, moderately diverging posteriorly, moderately convex at posterolateral parts of disc, legs orange at pro-coxae and trochanters. Abdominal sternite VIII (Fig. 8E) slightly protuberant on both sides of posterior margin, latero-apical angles subrounded. Internal organ of reproductive system (Fig. 10B): vagina stout and abruptly narrowed and extended into a long duct above median oviduct; diverticulum and spermathecal duct arising from the end of the long duct of vagina; diverticulum slightly long, thin and spiral; spermathecal duct distinctly thicker and slightly shorter than diverticulum; spermatheca composed of a spiral tube which is distinctly longer than diverticulum, provided with a very long and thin accessory gland (surrounded with a slightly sclerotized sheath, which is hard to be stripped) which is slightly longer than the spiral tube of spermatheca; median oviduct situated in middle of vagina.

Body length: 6.5‒9.0 mm; width: 1.2‒1.8 mm.

##### Diagnosis.

This species is similar to *Fissocantharis gracilipes* (Pic, 1927), but differs in the smaller body; aedeagus: conjoint dorsal plate of parameres moderately reduced, ventral process of each paramere evenly narrowed apically, moderately hooked at apex.

##### Distribution.

China (Guangxi).

##### Etymology.

The specific name is derived from Latin *eschara* (scar), referring its antennomeres IV‒XI with scar-like bulges along the outer margins in male.

##### Remarks.

Sometimes the pronotum presents with a large inverse-triangular and a slightly small triangular black marking in middle of anterior and posterior parts of disc respectively, which are almost conjoint.

#### Fissocantharis latipalpa

Taxon classificationAnimaliaColeopteraCantharidae

Y. Yang & X. Yang sp. n.

http://zoobank.org/9457F8A6-3CB0-4F3F-9366-343F417EA593

**Figures 6.**
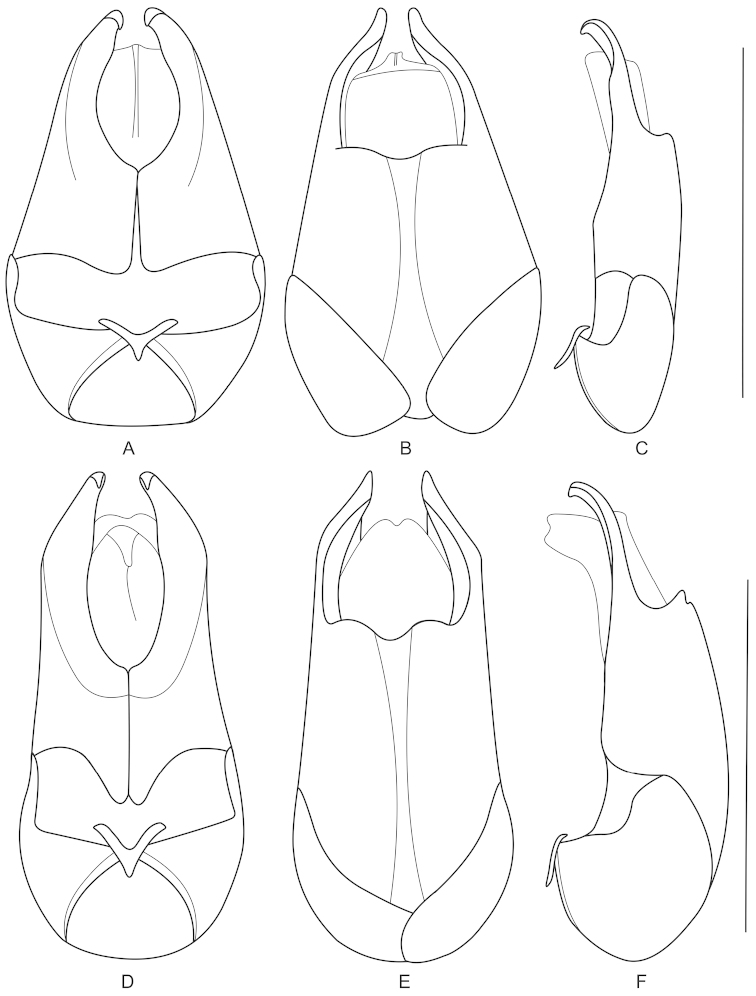
Aedeagus (**A, D** ventral view **B, E** dorsal view **C, F** lateral view): **A–C** *Fissocantharis latipalpa* sp. n. **D–F** *Fissocantharis biprojicientis* sp. n. Scale bars: 1.0 mm.

Figs 2C , 6A‒C , 8F , 10C

##### Type material.

Holotype ♂ (MHBU): CHINA: Guangxi, Mao’ershan, 1235m, 2.VI.2011, leg. H.Y. Liu. Paratypes: CHINA: Guangxi: 3♀♀ (MHBU): same data as the holotype.

##### Description.

Male (Fig. 2C). Head yellow, mouthparts yellow, dark brown at apices of mandibles, antennae yellow, slightly darkened at antennomeres XI, pronotum, scultellum and elytra black, legs yellow, slightly darkened at tarsomeres IV‒V, presternum yellow, meso- and metasterna black, abdomen black, light yellow at posterior margins of all visible abdominal sternites and apical half of IX. Body densely covered with short decumbent light yellow pubescence, also mixed with slightly long semierect pubescence along anterior margin of labrum and on disc of elytra.

Head subquadrate, temples evenly narrowed posteriad, surface semilustrous, finely and sparsely punctate; eyes slightly protruding, head breadth across eyes distinctly wider than anterior margin of pronotum; maxillary palpomeres II‒IV distinctly flattened and widened, II mountain-shapely convex at outer parts of dorsal sides, III wider than long, slightly widened apically, IV longer than wide, distinctly narrowed apically, with outer margin arcuate and sharp at apical part; antennae extending to elytral mid-length, antennomeres II nearly as long as wide at apices, III‒X slightly widened apically, III about 1.5 times as long as wide, IV about one-third longer than III, VI longest, XI slightly longer than X, nearly parallel-sided and pointed at apex.

Pronotum about 1.13 times longer than wide, anterior margin rounded, anterior angle rounded, lateral margins slightly diverging posteriorly, posteriad angle nearly rectangular, posterior margin arcuate and slightly bordered, disc moderately convex on postero-lateral parts, surface semilustrous, sparsely and finely punctate.

Elytra about 4.3 times longer than pronotum, 3.0 times longer than humeral width, lateral margins nearly parallel, disc surface semilustrous, rugulose-lacunose and finely punctate.

All tarsal claws bifid, upper claws nearly as long as lower claws.

Aedeagus (Fig. 6A‒C): conjoint dorsal plate of parameres greatly reduced, slightly roundly emarginated in middle of apical margin; ventral process of each paramere evenly narrowed apically, largely hooked at apex.

Female. Similar to male, but maxillary palpi normal; antennae shorter, extending to basal one-third length of elytra, antennomeres II about 1.5 times as long as wide at apices, III‒X parallel-sided; pronotum slightly wider, nearly as long as wide, slightly convex at postero-lateral parts of disc; elytra with lateral margins slightly diverging posteriad. Abdominal sternite VIII (Fig. 8F) slightly emarginated on both sides of posterior margin, middle part between lateral emarginations subtruncated, latero-apical angles widely rounded. Internal organ of reproductive system (Fig. 10C): vagina stout and abruptly narrowed and extended into a long duct above median oviduct; diverticulum and spermathecal duct arising from the end of the long duct of vagina; diverticulum moderately long, thin and spiral; spermathecal duct distinctly thicker and shorter than diverticulum; spermatheca composed of a spiral tube which is nearly as long as diverticulum, provided with a moderately long and thin accessory gland, which is nearly as long as the spiral tube of spermatheca; median oviduct situated in middle of vagina.

Body length: 6.5‒7.5 mm; width: 1.3‒1.5 mm.

##### Diagnosis.

This species is similar to *Fissocantharis pallidiceps* (Pic, 1911), but can be easily distinguished from the latter by the characteristic maxillary palpi in the male, of which palpomeres II‒IV are flattened and widened; aedeagus: conjoint dorsal plate of parameres greatly reduced, slightly emarginated in middle of apical margin.

##### Distribution.

China (Guangxi).

##### Etymology.

The specific name is derived from Latin *latus* (wide) and *palpus* (palp), referring to its maxillary palpomeres II‒IV flattened and widened in male.

#### Fissocantharis biprojicientis

Taxon classificationAnimaliaColeopteraCantharidae

Y. Yang & X. Yang sp. n.

http://zoobank.org/5F4FCF35-FB3C-4A5D-BE0F-65D6CB409F53

**Figure 7.**
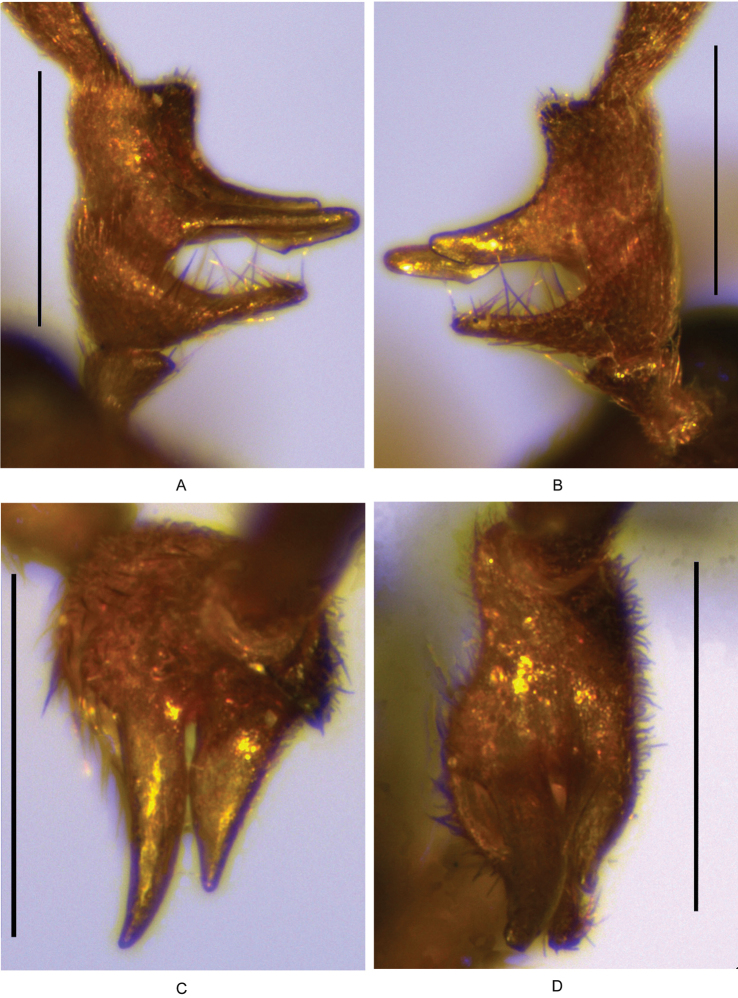
Male antennomeres III–IV of *Fissocantharis biprojicientis* sp. n.: **A** ventral view **B** dorsal view **C** apical view **D** ventroapical view. Scale bars: 1.0 mm.

**Figure 8.**
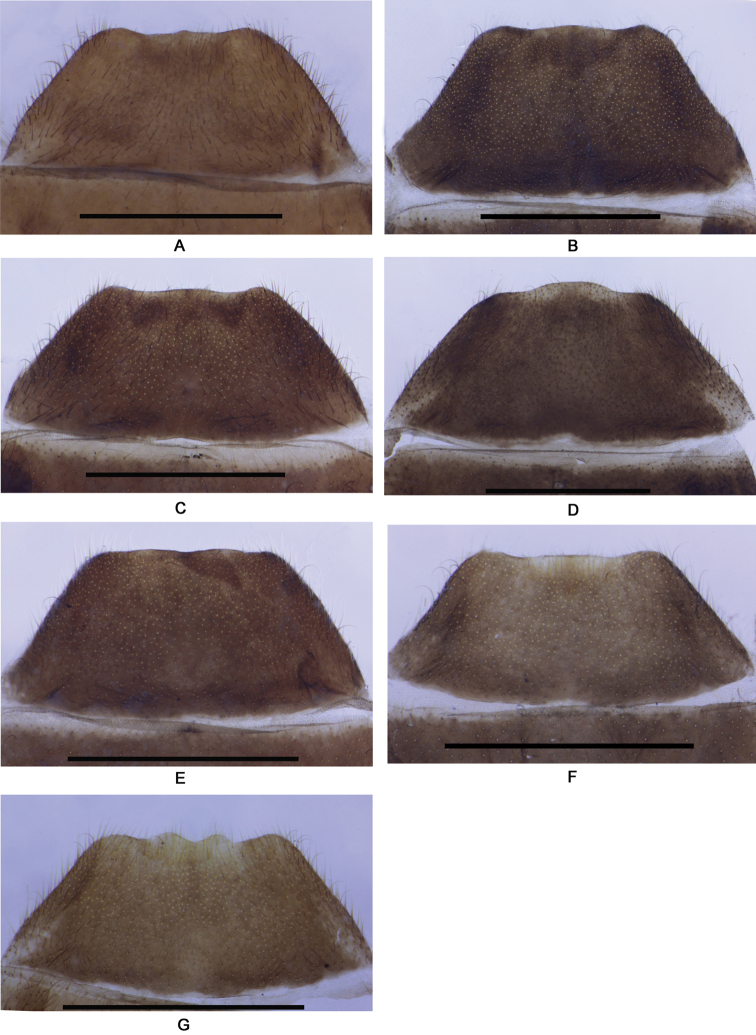
Abdominal sternite VIII of female, ventral view: **A** *Fissocantharis sinensis* (Wittmer, 1988) **B** *Fissocantharis gracilipes* (Pic, 1927) **C** *Fissocantharis sexcostata* sp. n. **D** *Fissocantharis basilaris* sp. n. **E** *Fissocantharis eschara* sp. n. **F** *Fissocantharis latipalpa* sp. n. **G** *Fissocantharis biprojicientis* sp. n. Scale bars: 1.0 mm.

Figs 2D , 6D‒F , 7 , 8G , 10D

##### Type material.

Holotype ♂ (IZAS): CHINA: Guangxi, Jinxiu, Rd. Jinzhong, 1100m, 10.V.1999, leg. D.C. Yuan. Paratypes: CHINA: Guangxi: 1♀ (IZAS): Jinxiu, Shengtangshan, 700‒800m, 19.V.1999, leg. H. Xiao. 1♀ (IZAS): same locality, 900‒1900m, 17.V.1999, leg. H.X. Han; 1♀ (IZAS): Yonghe, 500m, 11.V.1999, leg. H. Xiao.

##### Description.

Male (Fig. 2D). Head and mouthparts orange, dark brown at apices of mandibles, terminal maxillary and labial palpomeres and antennae black, antennomeres I‒IV and basal parts of V, prothorax and legs orange, darkened at tarsomeres II‒V, the rest parts of body black. Body densely covered with short decumbent light orange pubescence, also mixed with slightly long semierect pubescence along anterior margin of labrum and on disc of elytra.

Head subquadrate, temples evenly narrowed posteriad, surface semilustrous, finely and sparsely punctate; eyes slightly protruding, head breadth across eyes distinctly wider than anterior margin of pronotum; maxillary palpomeres II‒III excavated wholly on dorsal sides, IV longer than wide, nearly parallel-sided, arcuate and sharp at apices; antennae (Fig. 7) extending to elytral mid-length, antennomeres II short, about twice wider than long, III strongly widened apically, with outer-apical angle distinctly projecting laterad, IV thickened and excavated at ventral sides, with two long and pointed projections at basal parts, dorsal projections slightly shorter than ventral ones, which are triangularly protuberant at lower margins near apices, V‒X slightly widened apically, XI slightly shorter than X and pointed at apices.

Pronotum about 1.22 times longer than wide, anterior margin rounded, anterior angle distinctly rounded, lateral margins nearly parallel, posterior angle nearly rectangular, posterior margin arcuate and slightly bordered, disc moderately convex on postero-lateral parts, surface semilustrous, sparsely and finely punctate.

Elytra about 4.3 times longer than pronotum, 3.5 times longer than humeral width, lateral margins nearly parallel, disc surface semilustrous, rugulose-lacunose and finely punctate.

All tarsal claws bifid, upper claws nearly as long as lower claws.

Aedeagus (Figs 6D‒F): conjoint dorsal plate of parameres greatly reduced, roundly emarginated in middle of apical margin; ventral process of each paramere evenly narrowed apically at apical part, which distinctly narrower than basal part, moderately hooked at apex.

Female. Similar to male, but maxillary palpi normal; antennae orange at antennomeres I‒III and bases of IV, II about twice longer than wide, III‒IV normal; pronotum slightly wider, about 1.12 times longer than wide, slightly convex at postero-lateral parts of disc. Abdominal sternite VIII (Fig. 8G) triangularly emarginated on both sides and roundly emarginated in middle of posterior margin, the parts between lateral and middle emarginations subrounded at apices, latero-apical angles widely rounded. Internal organ of reproductive system (Fig. 10D): vagina stout and abruptly narrowed and extended into a long duct above median oviduct; diverticulum and spermathecal duct arising from the end of the long duct of vagina; diverticulum moderately long, thin and spiral; spermathecal duct distinctly thicker and shorter than diverticulum; spermatheca composed of a spiral tube which is slightly shorter than diverticulum, provided with a moderately long and thin accessory gland, which is slightly shorter than the spiral tube of spermatheca; median oviduct situated in middle of vagina.

Body length: 7.0‒9.0 mm; width: 1.5‒1.8 mm.

**Figure 9.**
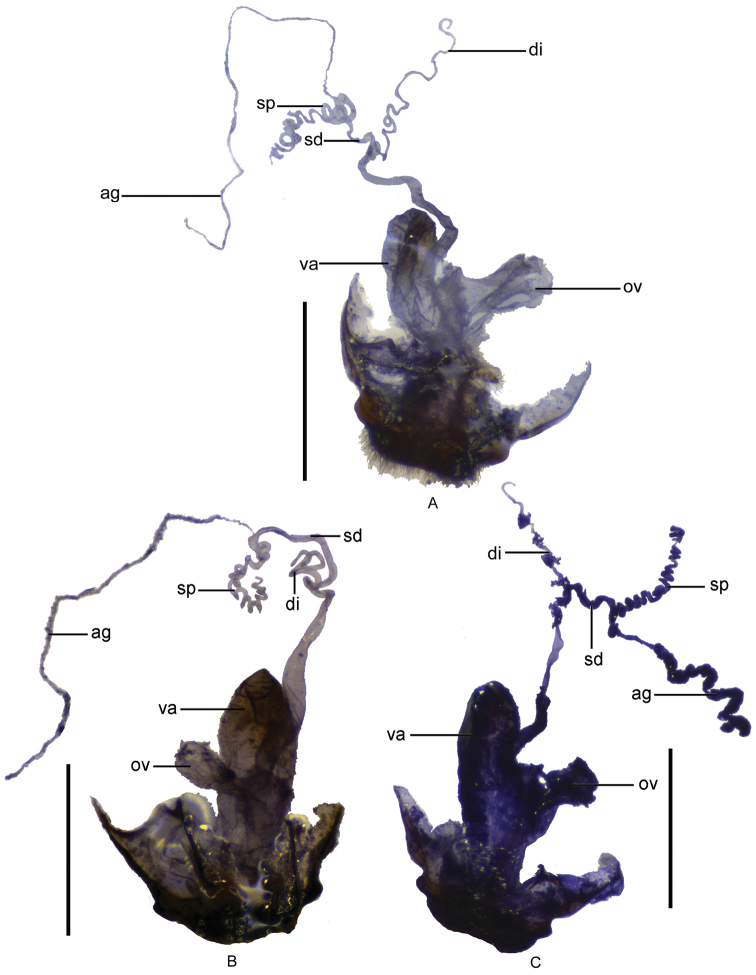
Female genitalia: **A** *Fissocantharis sinensis* (Wittmer, 1988) **B** *Fissocantharis gracilipes* (Pic, 1927) **C** *Fissocantharis sexcostata* sp. n. Scale bars: 1.0 mm.

**Figure 10.**
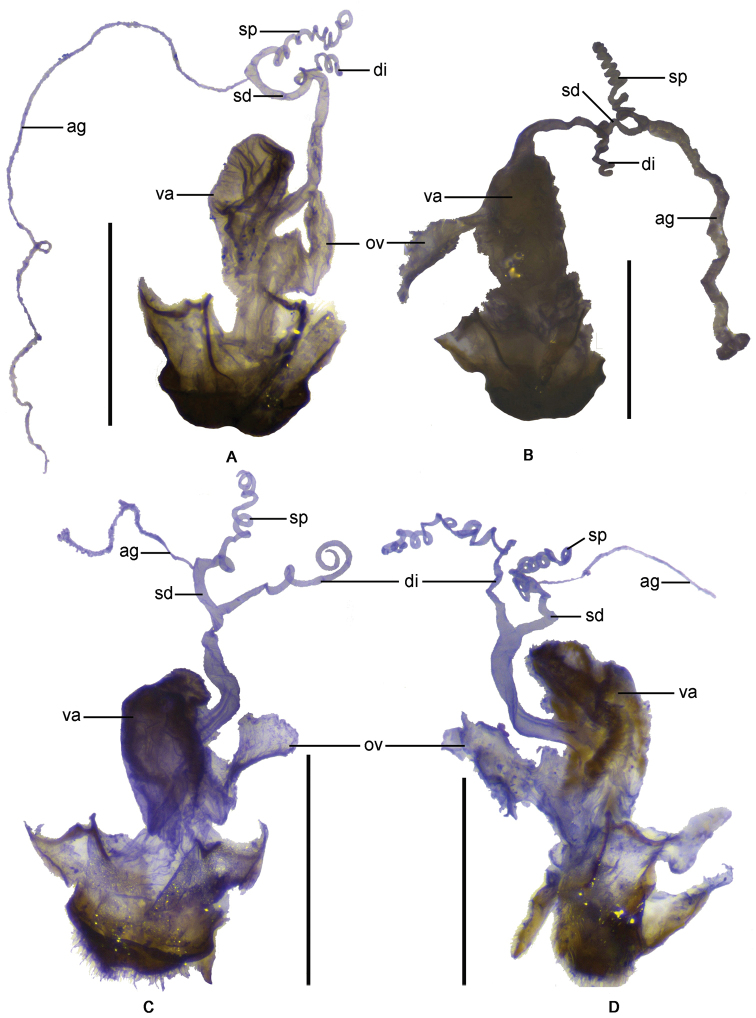
Female genitalia: **A** *Fissocantharis basilaris* sp. n. **B** *Fissocantharis eschara* sp. n. **C** *Fissocantharis latipalpa* sp. n. **D** *Fissocantharis biprojicientis* sp. n. Scale bars: 1.0 mm.

##### Diagnosis.

This species is similar to *Fissocantharis bidifformis* (Wittmer, 1988), but it can be differentiated from the latter by the antennomeres IV with two projections on the basal part in the male; aedeagus: conjoint dorsal plate of parameres greatly reduced, roundly emarginated in middle of apical margin.

##### Distribution.

China (Guangxi).

##### Etymology.

The specific name is derived from the suffix *bi*- (two) and *projicientis* (projecting), referring to its antennomere IV with two projections on the basal part in the male.

### Other species of *Fissocantharis* known from Guangxi, China

#### Fissocantharis angusta

Taxon classificationAnimaliaColeopteraCantharidae

(Fairmaire, 1900)

Podabrus angustus Fairmaire, 1900: 624.
Podabrus flavofacialis Pic, 1926: 29. **syn. n.**
Podabrus denticornis Wittmer, 1951: 96, fig. 2. Synonymized with *Podabrus flavofacialis* Pic by Wittmer 1988: 357.
Micropodabrus angustus : Wittmer 1988: 344.
Micropodabrus flavofacialis : Wittmer 1988: 357.
Fissocantharis angusta : Yang et al. 2009: 49.
Fissocantharis flavofacialis : Yang et al. 2009: 49.

##### Type material examined.

*Podabrus angustus*: Holotype: 1♀ (MNHN): “Fokien” [China: Fujian], “Podabrus \ angustus \ Fairm. China”, “Micropodabrus \ angustus \ (Fairm.) \ det. W. Wittmer”, “HOLOTYPUS”. Paratypes: 1♂, 1♀ (MNHN): de Latouche, 1900, H. Donckier.

*Podabrus flavofacialis*: Holotype: 1♂ (MNHN): “Fokien”, “flavofacialis \ Pic”, “Micropodabrus \ flavofacialis \ (Pic) \ det. W. Wittmer”, “HOLOTYPUS”.

*Podabrus denticornis*: Holotype: 1♂ (ZFMK): “Kuatun (2300m) 27.40n.Br.\117.40ö.L. J. Klappperich \ 28.5.1938 (Fukien)”, “Holotypus \ Podabrus \ denticornis \ Wittmer 49, n. sp.”, “Podabrus \ denticornis \ Wittm.”, “Micropodabrus \ flavofacialis \ (Pic) \ det. W. Wittmer”, “MUSEUM KOENIG \ BONN”. Paratypes: 1♀ (MNHN): same data, 12.5.1938; 1♀ (MNHN): same data, 19.5.1938; 1♀ (MNHN): same data, 20.5.1938; 1♀ (MNHN): same data, 28.5.1938; 1♂, 1♀ (NHMB): same data, 18.5.1938.

##### Additional material examined.

CHINA: Zhejiang: 2♀♀ (MNHN): Tienmushan, 9.VI.1936, coll. O. Piel; 1♀ (IZAS): Tienmushan, 6.VI.1936, coll. O. Piel; 1♀ (IZAS): Tienmushan, 12.VI.1936; 1♀ (IZAS): Tienmushan, 6.VI.1936; 1♂ (IZAS): Tienmushan, 8.VI.1936; 1♂ (IZAS): Anji, Longwangshan, 500m, 11.VI.1996, leg. X.K. Yang; 1♂, 1♀ (IZAS): same locality and date, leg. W.Z. Li; 1♀ (IZAS): same locality and collector, 12.VI.1996; 1♂, 1♀ (IZAS): same locality and collector, 13.VI.1996. Hunan: 1♂, 3♀♀ (NHMB): Wulingshan, Tianzishan Nat. Res., 800m, 16.‒18.VI.1997, lgt. Bolm. Guangxi: 1♂ (IZAS): Jinxiu, Rd. Jinzhong, 1000m, 12.V.1999, leg. M.Y. Gao; 1♀ (IZAS): same locality and date, 1100m, leg. X.K. Yang; 2♂♂, 4♀♀ (IZAS): same locality and date, leg. H. Xiao; 1♂ (IZAS): same locality, 11.V.1999, leg. D.C. Yuan; 1♀ (IZAS): Jiuxiu, Yonghe, 500m, 11.V.1999, leg. H. Xiao; 1♀ (IZAS): same locality and date, leg. F.S. Huang; 1♀ (IZAS): Jinjiu, Shengtangshan, 700‒800m, 19.V.1999, leg. H. Xiao.

##### Distribution.

China (Fujian, Zhejiang, Hunan, Guangxi). Newly record for Zhejiang, Hunan and Guangxi, China.

##### Remarks.

Based on the examination of the types, *Fissocantharis flavofacialis* (Pic, 1926) is considered to be a junior synonym of *Fissocantharis angusta* (Fairmaire, 1900). Although the holotype of the latter species is female and the former is male, both species are originally described in *Podabrus* Westwood and attached with the same locality labels; also a large number of additional specimens do not show any difference between them. Therefore, we suggest to synonymize *Fissocantharis flavofacialis* with *Fissocantharis angusta*.

#### Fissocantharis bidifformis

Taxon classificationAnimaliaColeopteraCantharidae

(Wittmer, 1988)

Micropodabrus bidifformis Wittmer, 1988: 350, Figs 4, 23.
Fissocantharis bidifformis : Yang et al. 2009: 49.

##### Material examined.

CHINA: Guangdong: 1♂ (SYSU): Lianxian, Dadongshan, 27.V.1997, leg. X.X. Zhang; 1♀ (SYSU): same locality, 28.V.1997, leg. J.H. Li; 1♀ (SYSU): same locality, leg. J. Zheng.

##### Distribution.

China (Guangxi, Guangdong). Newly record for Guangdong, China.

#### Fissocantharis buonloiensis

Taxon classificationAnimaliaColeopteraCantharidae

Wittmer, 1993

Micropodabrus buonloiensis Wittmer, 1993: 217, Figs 22, 26.
Fissocantharis buonloiensis : Yang et al. 2009: 49.

##### Distribution.

China (Guangxi); Vietnam.

#### Fissocantharis cicatricosa

Taxon classificationAnimaliaColeopteraCantharidae

(Wittmer, 1988)

Micropodabrus cicatricosus Wittmer, 1988: 360, Figs 14, 33.
Fissocantharis cicatricosa : Yang et al. 2009: 49.

##### Distribution.

China (Fujian, Guangxi).

#### Fissocantharis flavicornis

Taxon classificationAnimaliaColeopteraCantharidae

(Gorham, 1889)

Telephorus flavicornis Gorham, 1889: 108.
Cantharis flavicornis : Jacobson 1911: 679.
Podabrus flavicornis : Wittmer 1969: 131.
Micropodabrus flavicornis : Wittmer 1988: 360.
Fissocantharis flavicornis : Yang et al. 2009: 49.

##### Material examined.

CHINA: Guizhou: 4♂♂, 4♀♀ (NHMB): Dakua, 35km NE Leishan, 20.‒24.VI.1994, lgt. Bolm.

##### Distribution.

China (Fujian, Guangxi, Guizhou). Newly record for Guizhou, China.

#### Fissocantharis liuchowensis

Taxon classificationAnimaliaColeopteraCantharidae

(Wittmer, 1989)

Micropodabrus liuchowensis Wittmer, 1989: 212, Figs 8, 9.
Fissocantharis liuchowensis : Yang et al. 2009: 49.

##### Distribution.

China (Guangxi).

#### Fissocantharis multiexcavata

Taxon classificationAnimaliaColeopteraCantharidae

(Wittmer, 1988)

Micropodabrus multiexcavatus Wittmer, 1988: 361, Figs 16, 34.
Fissocantharis multiexcavata : Yang et al. 2009: 49.

##### Distribution.

China (Guangxi); Vietnam.

#### Fissocantharis tachulanensis

Taxon classificationAnimaliaColeopteraCantharidae

(Wittmer, 1988)

Micropodabrus tachulanensis Wittmer, 1988: 358, Figs 12, 32.
Fissocantharis tachulanensis : Yang et al. 2009: 49.

##### Distribution.

China (Fujian, Guangxi).

#### Fissocantharis tridifformis

Taxon classificationAnimaliaColeopteraCantharidae

(Wittmer, 1988)

Micropodabrus tridifformis Wittmer, 1988: 349, Figs 2, 21.
Fissocantharis tridifformis : Yang et al. 2009: 49.

##### Material examined.

CHINA: Hubei: 1♂, 1♀ (IZAS): Shennongjia, 900‒1300m, 23.V.1981, leg. Y.H. Han; 1♀ (IZAS): same locality and collector, 900‒1700m, 26.V.1981; 1♂ (IZAS): same locality and collector, 900m, 16.VI.1981.

##### Distribution.

China (Sichuan, Guangxi, Hubei). Newly recorded from Hubei, China.

## Supplementary Material

XML Treatment for Fissocantharis sinensis

XML Treatment for Fissocantharis gracilipes

XML Treatment for Fissocantharis sinensomima

XML Treatment for Fissocantharis sexcostata

XML Treatment for Fissocantharis basilaris

XML Treatment for Fissocantharis eschara

XML Treatment for Fissocantharis latipalpa

XML Treatment for Fissocantharis biprojicientis

XML Treatment for Fissocantharis angusta

XML Treatment for Fissocantharis bidifformis

XML Treatment for Fissocantharis buonloiensis

XML Treatment for Fissocantharis cicatricosa

XML Treatment for Fissocantharis flavicornis

XML Treatment for Fissocantharis liuchowensis

XML Treatment for Fissocantharis multiexcavata

XML Treatment for Fissocantharis tachulanensis

XML Treatment for Fissocantharis tridifformis
